# Thoraco-abdominal normothermic regional perfusion does not restore cerebral blood flow or electrical activity despite collateral supra-aortic blood flow in a porcine model

**DOI:** 10.1016/j.jhlto.2025.100221

**Published:** 2025-01-29

**Authors:** Matthieu Glorion, Joel Neves Briard, Louise Roquebert, Sabina Pizzi, Ahmed Menaouar, Mélanie Borie, Manon Robert, Dang Khoa Nguyen, Michaël Chassé, Basil Nasir, Pasquale Ferraro, Shant Der Sarkissian, Pierre-Emmanuel Noly, Nicolas Noiseux

**Affiliations:** a Centre de Recherche du Centre Hospitalier de l′Université de Montréal (CRCHUM), Montréal, Québec, Canada; b Division of Thoracic Surgery, Department of Surgery, Université de Montréal, Montréal, Québec, Canada; c Service de Chirurgie Thoracique, Hôpital Foch, Suresnes, France; d Department of Neuroscience, Université de Montréal, Montréal, Québec, Canada; e Department of Medicine, Université de Montréal, Montréal, Québec, Canada; f Department of Surgery, Université de Montréal, Montréal, Québec, Canada; g Division of Cardiac Surgery, Department of Surgery, Université de Montréal, Montréal, Québec, Canada

**Keywords:** organ donation, cardiocirculatory death, animal model, electroencephalography, angiography, thoraco-abdominal normothermic regional perfusion (TA-NRP)

## Abstract

**Background:**

It is unknown whether ligation of supra-aortic vessels during thoraco-abdominal normothermic regional perfusion (TA-NRP) can prevent postmortem brain function, ensuring the permanence of death. Our objective was to determine if ligation of the supra-aortic vessels during TA-NRP prevents resumption of intracranial blood flow, brain electrical activity and clinical brain function in a porcine model of organ donation after circulatory arrest.

**Methods:**

Neuromonitoring was performed in 9 porcine experiments, in which supra-aortic vessels were ligated.

**Results:**

During TA-NRP and organ procurement, no motor reaction to pain, spontaneous ventilation, eye movement or change in pupillary function were observed. Angiography demonstrated absence of supra-aortic blood flow in 4 (44%) experiments and delayed, discrete and transient supra-aortic extracranial opacification in 5 (56%) experiments. No intracranial blood flow was observed. All electroencephalograms demonstrated absent brain electrical activity.

**Conclusion:**

In this porcine model, occlusion of the supra-aortic vessels during TA-NRP did not restore post-mortem cerebral blood flow or electrical activity and is in adequation with the permanence of death.

In the context of controlled organ donation following circulatory arrest, thoraco-abdominal normothermic regional perfusion (TA-NRP) has numerous advantages for organ procurement, including heart retrieval for transplantation. Since death determination relies on the permanent cessation of brain function, post-mortem interventions that restore systemic blood flow and perfusion in donors, such as TA-NRP, must ensure that brain function cannot resume.^1^, ^2^, ^3^ Although supra-aortic blood vessels are clamped, ligated, or sectioned during TA-NRP, collateral blood flow through thoracic and spinal vasculature could theoretically permit resumption of cerebral blood flow.^4^ It is currently unknown whether TA-NRP collateral blood flow can restore brain perfusion and function, which would violate the dead donor rule and preclude organ donation after circulatory arrest.^1^ Our objective was to determine if supra-aortic vessel ligation during TA-NRP prevents resumption of intracranial blood flow, brain electrical activity and clinical brain function in a porcine model of organ donation after circulatory arrest.

## Material and methods

**Figure 1:**
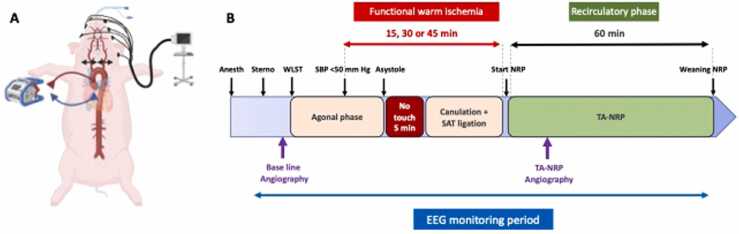
Experimental model. Anesth, general anesthesia; EEG, electroencephalography; NRP, normothermic regional perfusion; SAT, supra-aortic trunks; SBP, systolic blood pressure; Sterno, median sternotomy; TA-NRP, thoraco-abdominal normothermic regional perfusion; WLST, withdrawal of life-sustaining therapies.

This project was approved by the Institutional Review Board of the Center hospitalier de l′Université de Montréal (#2022-10333). All animals received humane care in compliance with the Guide for the Care and Use of Laboratory Animals.^5^ Nine female white hybrid pigs (55–65 kg, Ferme Triporc, Canada) were allocated to 1 of 3 groups with different functional warm ischemic times (f-WIT): 15 minutes (*n* = 3), 30 minutes (*n* = 3), and 45 minutes (*n* = 3). f-WIT was defined as the time from when systolic blood pressure fell below 50 mm Hg to TA-NRP onset (Figure 1).

The pigs were anesthetized using a continuous intravenous anesthesia protocol (propofol [Fresenius Kabi Canada] 6–20 mg/kg/hour, fentanyl [Fresenius Kabi Canada] 20–50 µg/kg/hour, ketamine [Sandoz Canada] 0,3 mg/kg/hour) and paralyzed with rocuronium until withdrawal of life-sustaining therapies. Central femoral venous and arterial lines were placed under ultrasound guidance. Mechanical ventilation was initiated in volume control mode, set to a respiratory rate of 20 breaths/min, 7 ml/kg of tidal volume, 5 cm H_2_O positive end-expiratory pressure and a fraction of inspired oxygen at 40%. Following median sternotomy to expose the heart and great vessels, a cannula was inserted into the ascending aorta (dual lumen aortic root cannula vent 2 mm Medtronic) for angiography.

After administering heparin (Sandoz Canada) at 500 IU/kg and an intravenous bolus of propofol (3 mg/kg) for pig comfort, hypoxic circulatory arrest was induced by discontinuing mechanical ventilation via clamping of the endotracheal tube under general anesthesia (withdrawal of life-sustaining therapies). At this point, all hypnotic, analgesic, and paralytic drugs were discontinued. After asystole, determined by the loss of pulse pressure on the arterial waveform, a 5-minute stand-off period was observed before proceeding with TA-NRP cannulation.

Central TA-NRP was achieved through cannulation of the aorta and right atrium with systematic left atrium venting.^6^ Both supra-aortic vessels were ligated with sutures at emergence from the aorta prior to TA-NRP as recommended in the technical standards for TA-NRP set forth by the American Society of Transplant Surgeons, the International Society of Heart and Lung Transplantation, the Society of Thoracic Surgeons and the American Association for Thoracic Surgery.^7^ Mechanical ventilation was restarted at the same time of reperfusion. A standard cardiopulmonary bypass circuit comprising a membrane oxygenator, centrifugal pump, hardshell reservoir, and heat exchanger was used for TA-NRP. Oxygenated normothermic blood was delivered into the ascending aorta, with a target mean arterial pressure of 55 mm Hg, using vasopressors as needed. Defibrillation was attempted when acid-base and electrolyte balance was achieved. TA-NRP was thereafter weaned prior to organ procurement.

Neuromonitoring included clinical observation, conventional angiography and continuous electroencephalography (EEG). A veterinary anesthetist monitored the pigs’ vital and neurologic signs throughout the duration of the intervention, noting any clinically detectible brain function (motor reaction to pain, spontaneous ventilation, eye movement and change in pupillary function). Angiographies (Siemens Artis-Q) were obtained by injecting 70 cc (10 cc/second) of iodine contrast into the aortic cannula and capturing fluoroscopic images during sufficient time for arterial and venous opacification to occur sequentially. Angiographies were acquired at 2 occasions during each experiment: at baseline and during TA-NRP. To ensure no anatomical variations were overlooked, we systematically compared the reference baseline angiography for each animal with the angiography performed after ligation of the supra-aortic trunks during TA-NRP. Based on previous work on porcine neurophysiology, a registered electrophysiology technician recorded continuous EEG throughout the duration of the experiment using subcutaneous electrodes placed at F3, F4, C3, C4, O1 and O2, with a Fz reference and 2 µV/mm sensitivity (BrainAmp, Brain Products).^8^

A neurologist and clinical neurophysiologist interpreted angiographies and EEG tracings. Angiographies were digitally substracted and evaluated for supra-aortic vessel opacification, noting its location and temporal evolution when present. American Clinical Neurophysiological Society criteria to determine death by neurologic criteria (i.e. isoelectric EEG) were applied to the EEG recordings, collecting the time at which the tracings became isoelectric. The neurophysiologist also recorded any cerebral activity or artefact occurring thereafter.^9^

## Results

**Figure 2:**
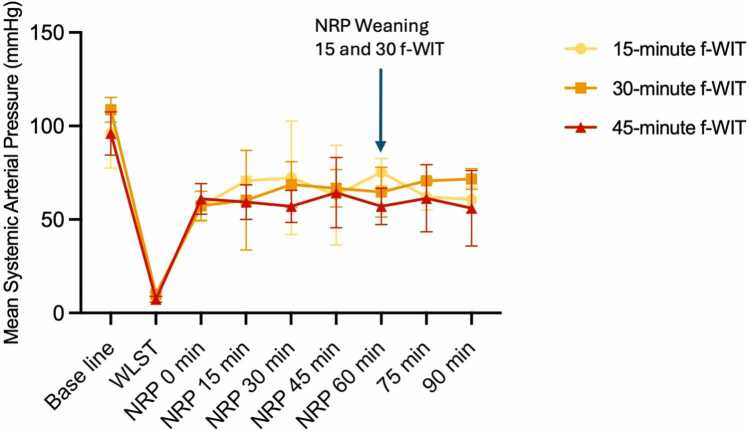
Evolution of the animals’ mean systemic arterial pressure over time. Mean systemic arterial pressure over time, measured via a femoral arterial catheter. f-WIT, functional warm ischemic time; NRP, normothermic regional perfusion; WLST, withdrawal of life-sustaining therapy.

Results of each experiment are provided in Table 1. Complete weaning from TA-NRP with cardiac activity evaluated off-pump for 30 minutes was achieved for all 3 pigs in the 15-minute f-WIT group and all 3 pigs in the 30-minute f-WIT group. In the 45-minute f-WIT group, no animals could be weaned from TA-NRP. The mean systemic arterial pressure during the TA-NRP period and after NRP weaning was consistently maintained above 55 mm Hg in all 3 groups (Figure 2).

**Table 1:** Neuromonitoring Results per Experiment

No	Ischemia time	Weaning from TA-NRP after 1 hour	Brain function during TA-NRP^a^	TA-NRP angiography	Isoelectric EEG during TA-NRP	Delay from WLST to isoelectric EEG (seconds)
1	45 minutes	No	No	Discrete delayed opacification of carotid arteries	Yes	173
2	45 minutes	No	No	No flow	Yes	473
3	45 minutes	No	No	No flow	Yes	58
4	30 minutes	Yes	No	No flow	Yes	202
5	30 minutes	Yes	No	Discrete delayed opacification of carotid arteries	Yes	231
6	30 minutes	Yes	No	No flow	Yes	174
7	15 minutes	Yes	No	Discrete delayed opacification of superficial arteries	Yes	116
8	15 minutes	Yes	No	Discrete delayed opacification of carotid arteries	Yes	161
9	15 minutes	Yes	No	Discrete delayed opacification of superficial arteries	Yes	Unknown^b^

EEG, electroencephalography; TA-NRP, thoraco-abdominal normothermic regional perfusion; WLST, withdrawal of life-sustaining therapies.

a Clinically detectible brain function monitored by the veterinary anesthetist were motor reaction to pain, spontaneous ventilation, eye movement and change in pupillary function.

b EEG monitor battery failure occurred during WLST. Battery was replaced and monitoring resumed during the warm ischemia period.

**Figure 3:**
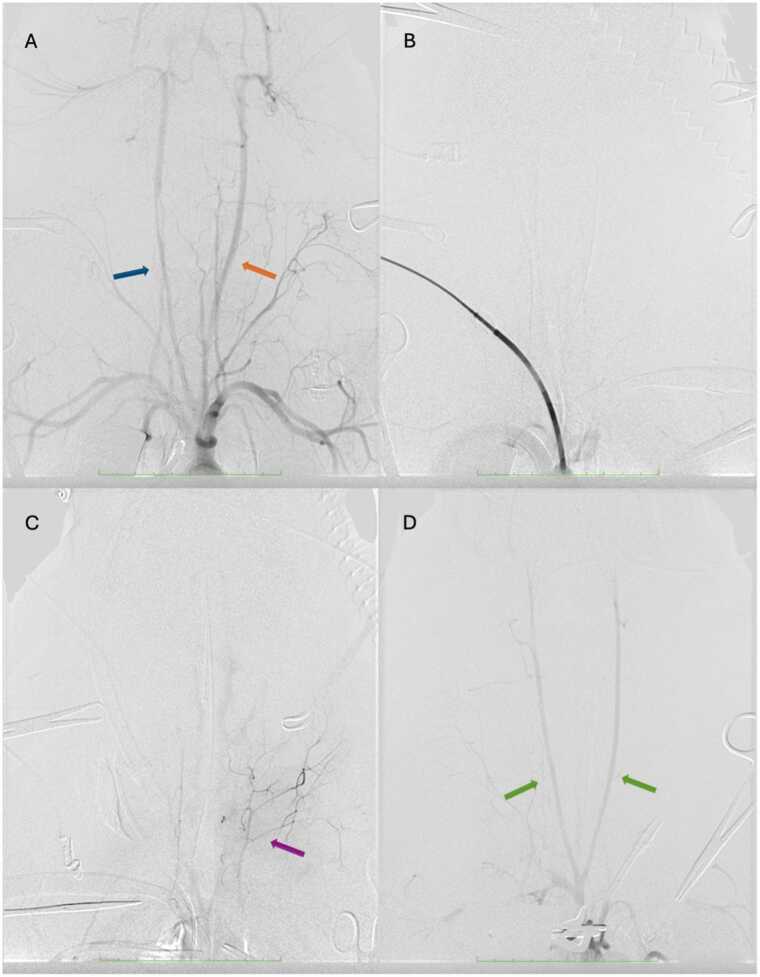
Digital substraction angiography findings. (A) Baseline angiography showing opacification of supra-aortic vessels, including left (orange arrow) and right (blue arrow) extracranial carotid arteries. (B) Thoraco-abdominal normothermic regional perfusion (TA-NRP) angiography showing no supra-aortic vessel opacification. (C) TA-NRP angiography showing opacification of superficial supra-aortic vessels (purple arrow). (D) TA-NRP showing opacification of the carotid arteries (green arrows).

**Figure 4:**
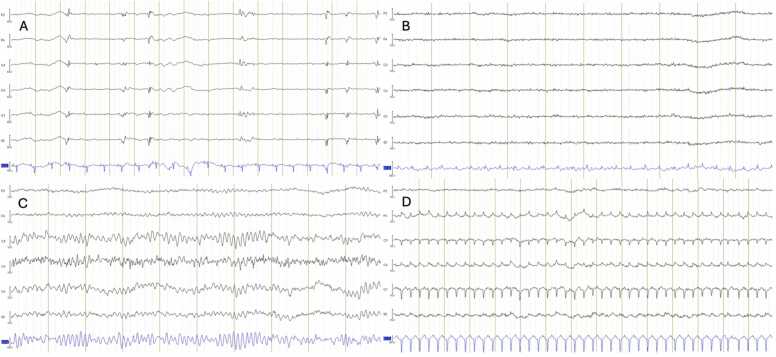
Electroencephalography findings. Referential montage, Fz reference, 60 Hz filter applied. Single-derivation electrocardiogram shown in blue. (A) Burst-suppression pattern, with inter-burst intervals of 1–3 seconds (B) No brain activity ≥2 µV is observed (i.e. isoelectric), which is consistent with the American Clinical Neurophysiology Society criteria for death determination by neurologic criteria. (C and D) Electrical artifact from ventricular fibrillation (C) and narrow QRS tachycardia (D) occurring during thoraco-abdominal normothermic regional perfusion.

After the agonal phase, when sedative, paralytic, and analgesic medications had been discontinued, no motor reaction to pain, no spontaneous ventilation, no eye movements and no changes in pupillary function were observed during clinical monitoring by the veterinary anesthetist. Angiography during TA-NRP demonstrated absence of supra-aortic blood flow in 4 (44%) experiments and presence of delayed, weak and transient extracranial supra-aortic opacification in 5 (56%) experiments (3 in the 15-minute f-WIT group, 1 in the 30-minute f-WIT group and 1 in the 45-minute f-WIT group; Figure 3 and Supplemental Figure 1). No intracranial blood flow was observed. All baseline EEGs showed continuous background activity. During circulatory arrest, periods of diffuse suppression progressed towards isoelectric EEG in all tracings (Figure 4). The median (interquartile range) delay between cessation of mechanical ventilation and isoelectric EEG was 174 (150−210) seconds. No EEG showed resumption of brain electrical activity during TA-NRP, but EEG frequently contained cardiac artifact during resuscitation (Figure 4).

## Discussion

In this porcine model of organ donation after circulatory arrest, we found that TA-NRP did not restore intracranial blood flow or electrical activity although delayed, discrete and transient supra-aortic, extracranial blood flow was documented in half of cases.

These findings support previous research showing that TA-NRP does not restore clinically relevant brain function or activity in experimental and clinical settings.^10^, ^11^, ^12^ However, since prior porcine neurophysiology experiments used single-lead EEG, spatial sampling was limited to a restricted region of the brain.^10^ Furthermore, these experiments used intradural electrodes, a monitoring modality that is unlikely to be subsequently translated to humans due to its invasiveness. In our work, we used EEG monitoring with conventional subcutaneous electrodes and sampled bilateral, anterior and posterior regions of the cerebrum, demonstrating robustly that occlusion of the supra-aortic vessels during TA-NRP is efficient to avoid thalamocortical cerebral activity in a porcine model of organ donation following circulatory arrest.

Supra-aortic blood flow is hypothesized to occur during TA-NRP due to collateral blood circulation through thoracic and spinal arteries.^4^ Since circulation does not infer perfusion or clinical function, the clinical significance of collateral blood flow for death permanence is currently unknown.^13^ Recently, a 2-case report of brain-injured human patients undergoing organ donation after circulatory arrest demonstrated absence of cerebral blood flow during intraoperative TA-NRP transcranial Doppler ultrasound monitoring, suggesting collateral blood flow, if present, is insufficient to perfuse the brain.^11^ In another case series involving 2 brain-injured individuals undergoing TA-NRP organ donation, invasive blood pressure monitoring in the middle cerebral arteries disclosed absence of pulsatile blood flow during the duration of the intervention.^12^ However, the impact of collateral blood flow on brain perfusion might depend on intracranial compliance, mean arterial pressure or other factors, such that these findings may not be generalizable to patients with normal intracranial pressure prior to circulatory arrest. In our study, collateral supra-aortic circulation was most commonly observed in the 15-minute f-WIT group, raising the hypothesis that more significant brain injury occurs as the duration of warm ischemia increases. Furthermore, the impact of collateral blood flow on brain perfusion might be influenced by the surgical technique employed to exclude supra-aortic circulation. In humans, transecting supra-aortic vessels and venting the cephalad ends to atmosphere have been proposed by some experts to prevent collateral blood flow to the brain.^4^, ^12^ However, since no studies have directly compared the effects of different surgical techniques on brain blood flow, perfusion or function, it is unclear whether venting cephalad ends to atmosphere is absolutely required to prevent brain perfusion or clinical function. In the 2 cases reported above, transcranial Doppler ultrasonography documented absence of brain blood flow despite supra-aortic vessels being clamped without transection or venting to atmosphere.^11^ In fact, current technical standards for TA-NRP suggest cerebral vessel management to occur with 1 of 4 techniques: vessel venting to atmosphere, vessel venting to NRP perfusion pump, vessel ligation with vascular stapler or vessel suture ligation, the latter of which was performed in our experiments.^7^

The strengths of our work include the use of angiographic, electroencephalographic and clinical neuromonitoring modalities to study complimentary elements of brain physiology during TA-NRP. Our work also has limitations. First, brainstem reflexes were not all formally monitored throughout TA-NRP. However, selected brainstem reflexes such as breathing and pupillary reaction were not observed. Second, although pigs share comparable anatomy to humans, our results do not guarantee that TA-NRP does not restore cerebral blood flow, electrical activity or clinical function in humans. For instance, ill adult humans who participate in controlled organ donation following circulatory arrest might have more robust collateral circulation than the healthy young pigs used in our experiments. Future work aiming to determine the impact of TA-NRP on the human brain should utilize rigorous multimodal neuromonitoring, ensuring that modalities are in place to assess blood flow (e.g. angiography, transcranial Doppler ultrasound), electrical activity (e.g. EEG, somatosensory evoked potentials) and clinical function (e.g. brainstem reflex assessment). Moreover, since the impact of TA-NRP on brain blood flow is likely dependent on the degree of brain injury prior to withdrawal of life sustaining therapies, further research is required to determine the effect of postmortem interventions that restore systemic circulation in different donor populations such as donors with severe brain injury and donors who undergo medical assistance in dying.

In conclusion, in a porcine model, occlusion of the supra-aortic vessels during TA-NRP did not restore post-mortem cerebral blood flow or electrical activity despite occasional delayed and transient supra-aortic blood flow and is in adequation with the permanence of death. Although these findings are not directly transposable to humans, they support prospective investigation on the impact of TA-NRP on human brain circulation, perfusion and clinical function.

## Author contributions

MG and JNB drafted the manuscript. JNB performed data analysis. All authors revised the manuscript for intellectual content and approved the final version of the manuscript.

## Disclosure statement

DKN is supported by the Canada Research Chair Program and declares financial support from Cambridge Press and Paladin Labs Inc. Other authors declare no competing interests.

## Acknowledgments

We would like to thank Sophie Grenon, Isabelle Houle, Alice Michalet Roy, Théoberte Lucien, Etienne Blais, for their precious help and support.

## Financial support

For this work, NN and PEN received funding through a Grant-in-Aid from the Heart and Stroke Foundation of Canada (G-23-0034193) and the Fonds Guy Roberge pour la recherche en maladies cardiovasculaires, as well as in-kind support from Medtronic. MG received fellowship funding from the Fondation pour la recherche en chirurgie thoracique de Montréal, Fondation Foch, and the Société Francophone de Transplantation. The funders had no role in study design, data collection and analysis, decision to publish, or preparation of the manuscript.

## Footnotes

Appendix A Supplementary data associated with this article can be found in the online version at doi:10.1016/j.jhlto.2025.100221.
