# Supplementary figures and images for: Thoraco-abdominal normothermic regional perfusion does not restore cerebral blood flow or electrical activity despite collateral supra-aortic blood flow in a porcine model
Source: JHLT Open. 2025 Jan 29;8:100221. doi: 10.1016/j.jhlto.2025.100221 (PMC11935451; doi:10.1016/j.jhlto.2025.100221)

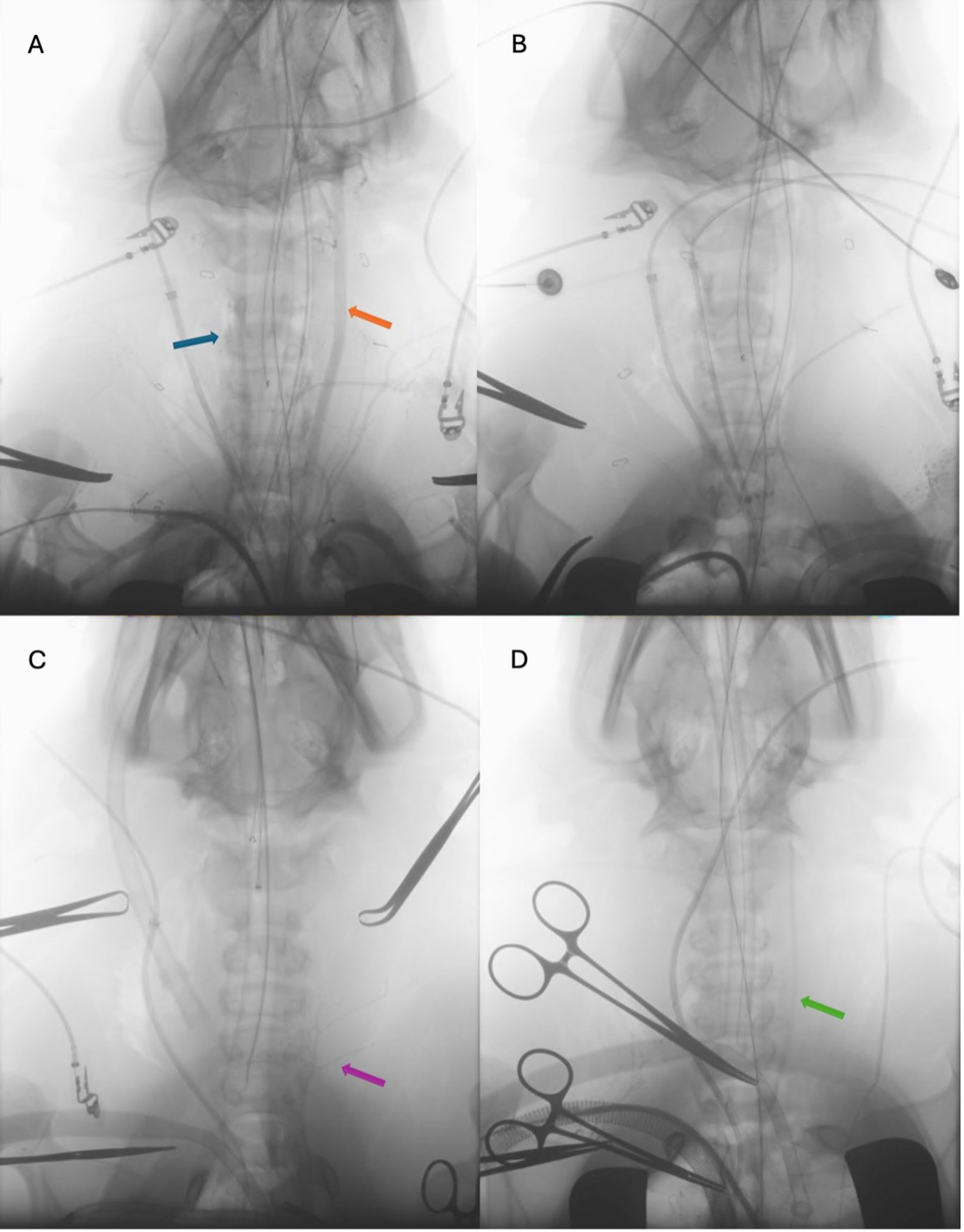

Supplement: Supplementary file 1 — Supplementary material [file mmc1.jpg]
